# Leptospirosis in Ecuador: Current Status and Future Prospects

**DOI:** 10.3390/tropicalmed8040202

**Published:** 2023-03-29

**Authors:** Manuel Calvopiña, Daniel Romero-Alvarez, Eduardo Vasconez, Gabriela Valverde-Muñoz, Gabriel Trueba, Miguel Angel Garcia-Bereguiain, Solon Alberto Orlando

**Affiliations:** 1 One Health Research Group, Facultad de Medicina, Universidad de Las Américas (UDLA), Quito 170124, Ecuador; 2 Biodiversity Institute and Department of Ecology and Evolutionary Biology, University of Kansas, Lawrence, KS 66045, USA; 3 Proyecto de Fortalecimiento de la Atención Integral de Personas con Discapacidad, Ministerio de Salud Pública, Quito 170702, Ecuador; 4 Institute of Microbiology, Universidad San Francisco de Quito, Quito 170901, Ecuador; 5 Universidad Latinoamerica de Costa Rica, San Jose 11501, Costa Rica; 6 Instituto Nacional de Investigación en Salud Pública INSPI, Quito 3961, Ecuador; 7 Universidad Espíritu Santo, Guayaquil 092301, Ecuador

**Keywords:** leptospirosis, *Leptospira*, zoonosis, neglected tropical disease, epidemiology, Ecuador

## Abstract

The location of Ecuador—an equatorial nation—favors the multiplication and dispersal of the *Leptospira* genus both on the Pacific Coast and in the Amazon tropical ecoregions. Nevertheless, leptospirosis epidemiology has not been fully addressed, even though the disease has been recognized as a significant public health problem in the country. The purpose of this literature review is to update knowledge on the epidemiology and geographical distribution of *Leptospira* spp. and leptospirosis in Ecuador to target future research and develop a national control strategy. A retrospective literature search using five international, regional, and national databases on *Leptospira* and leptospirosis including humans, animals, and environmental isolations of the bacteria and the disease incidence in Ecuador published between 1919 and 2022 (103 years) with no restriction on language or publication date was performed. We found and analyzed 47 publications including 22 of humans, 19 of animals, and two of the environments; three of these covered more than one of these topics, and one covered all three (i.e., One Health). Most (60%) of the studies were conducted in the Coastal ecoregion. Twenty-four (51%) were published in international journals, and 27 (57%) were in Spanish. A total of 7342 human and 6314 other animal cases were studied. Leptospirosis was a frequent cause of acute undifferentiated febrile illness in the Coast and Amazon and was associated with rainfall. All three major clusters of *Leptospira*—pathogenic, intermediate, and saprophytic—were identified from both healthy and febrile humans, the environment, and animals; moreover, nine species and 29 serovars were recorded over the three Ecuadorian ecoregions. *Leptospira* infections were diagnosed in livestock, companion, and wild animals from the Amazon and the Coast regions along with sea lions from the Galápagos Islands. Microscopic-agglutination test was the diagnostic tool most widely used. Three reviews covering national data on outpatients and inpatients determined the varied annual incidence and mortality rate, with males being more commonly affected. No human cases have been reported in the Galápagos Islands. Genomic sequences of three pathogenic *Leptospira* were reported. No studies on clinical ground, antibiotic resistance, or treatment were reported, nor were control programs or clinical-practice guidelines found. The published literature demonstrated that leptospirosis was and still is an endemic disease with active transmission in the four geoclimatic regions of Ecuador including the Galápagos Islands. Animal infections, distributed in mainland and insular Ecuador, pose a significant health risk for humans. Nationwide epidemiological surveys—encouraging more research on the fauna and environment with appropriate sampling design on risk factors for human and animal leptospirosis, *Leptospira* genotyping, increased laboratory capability, and readily available official data—are required to improve our understanding of transmission patterns and to develop effective national intervention strategies with the intention of applying One Health approaches.

## 1. Introduction

Leptospirosis—one of the most common and widespread zoonotic infections in the world—is caused by the Spirochaeta *Leptospira* and is recognized as a neglected communicable disease [1], particularly common in the tropical ecoregions of developing countries where people and animals live in close contact in areas where warm and humid conditions favor the environmental survival and transmission of the *Leptospira* species [2]. *Leptospira* spp. infecting human and other animal populations involve pathogenic, intermediate, and saprophytic clusters. The pathogenic is composed of nine species: *L. interrogans*, *L. kirschneri*, *L. noguchii*, *L. borgpetersenii*, *L. weilii*, *L. santarosai*, *L. alexanderi*, *L. kmetyi*, and *L. alstonii*. The intermediate contains six species, *L. fainei*, *L. licerasiae*, *L. inadai*, *L. broomii*, *L. idonii*, and *L. wolffii*, which associated with mild disease and chronic infections. The saprophytic cluster consists of seven species: *L. biflexa*, *L. terpstrae*, *L. meyeri*, *L. yanagawae*, *L. vanthielii*, *L parva*, and *L. wolbachii* [3]. Using molecular-genetic methods, Vincent et al. (2019) proposed a reclassification of the species of the *Leptospira* genus into four subclades—referred to as P1, P2, S1, and S2—instead of the clusters historically named as pathogens (P1), intermediates (P2), and saprophytes (S1 and S2) [4].

The severity of the disease depends on the infecting *Leptospira* spp. Symptoms in humans vary from none or a mild undifferentiated fever to a severe fulminating illness with acute kidney and liver failure or pulmonary hemorrhage; the fatality rates among confirmed cases range from 5% to 15% [3]. Globally, nearly 2.9 million disability-adjusted life years are lost annually due to leptospirosis [5]. The microscopic-agglutination test (MAT) has been considered the conventional technique for leptospirosis diagnosis [6,7] despite having many drawbacks—a sensitivity of only 80%, delayed results, unreliability, a low detection threshold, and difficult standardization—requiring trained personnel for culture and bacterial preservation [8,9]. MAT cannot discriminate between agglutinating antibodies occurring from current, recent, or past infection [6].

Ecuador, located in northwest South America, is currently considered, both nationally and internationally, endemic for leptospirosis with human outbreaks reported in urban, suburban, and rural populations along with infections in cattle, particularly in tropical ecoregions [6,7,10]. In this country, human leptospirosis is associated with a high number of cases as well as with high morbidity and fatality rates [6,11,12]. Veterinary studies, mainly in cattle, have revealed varying prevalence rates, ranging from 36% to 75% [10,13,14]. Concerns exist about animal leptospirosis and its impact on animal health, economic losses, and the spillover risk in the human–animal–ecosystem interface [10,15]. Despite this concern, no official document yet exists on the geographic distribution of *Leptospira* spp. in the environment, on human or animal disease, or on disease-control strategies.

Outbreaks occur during periods of heavy rainfall and flooding in urban slums when the environment and the water are contaminated by the urine of infected wild and/or domestic animals [16,17,18]. Ecuador is at particular risk of flooding during periods of intense rains that may be exacerbated by the El-Niño–Southern-Oscillation phenomenon (ENSO; [19]). The disability-adjusted lives associated with leptospirosis in Ecuador, estimated for a six-year period (2010–2015) were of US $152.83 per year along with an economic loss of US $988.727 per year at a total of US $5.9 million [18]. Nevertheless, despite the impact of the disease nationwide, large gaps exist in the knowledge of the burden and the epidemiology of leptospirosis in the country.

The first evidence of human leptospirosis in Ecuador was registered in 1918, when Hideyo Noguchi isolated *Leptospira* spp. from cases clinically diagnosed as yellow fever. Noguchi erroneously suggested that the etiologic agent of yellow fever was *Leptospira* [20]. A year later, Noguchi isolated *L. icterohaemorrhagiae* in rats and, based on immunological studies, subsequently differentiated between *L. icteroides* and *L. icterohaemorrhagiae* isolated from rats in Guayaquil city and elsewhere [21]. In Ecuador, the first bacteriologically confirmed human case caused by *L. icterohaemorrhagiae* was reported in 1924, although that species had been isolated from rats in Guayaquil in 1919 [22]. Other authors reported the isolation of *Leptospira* spp. in 1927, 1934, 1970, 1974, 1976, and 2013 from cases clinically diagnosed as leptospirosis, dengue, or hepatitis [23,24,25,26] as well as in rats of three different species and in *Didelphis marsupialis* from the Coastal provinces [27].

The Pan American Health Organization (PAHO) has estimated 10,702 cases of leptospirosis in the region annually, of which 7.2% are from Ecuador, the fourth highest prevalence after Brazil, Peru, and Colombia [7]. Internationally, Ecuador ranks 18 with a leptospirosis annual incidence of 11.6 per million people [28]. The Ecuadorian Ministry of Health (MoH) estimated an annual incidence of one case per 100,000 people at the national level, with 547 cases reported between 2016 and 2020, primarily from the Coastal provinces [11]. The most severe documented outbreak occurred in 1998 in the tropical city of Guayaquil where 80% of the cases required hospitalization, and 12% were fatal [17].

Despite the endemicity of leptospirosis in Ecuador, an official document considering the geographic distribution of the disease, reservoirs, and *Leptospira* spp. circulation is lacking. Moreover, clinical-practice guidelines or control strategies led by health authorities have not been instigated. Here, we review the available national and international literature regarding *Leptospira* spp. and leptospirosis in Ecuador to provide an updated synthesis of the available evidence on the ecology and epidemiology of the disease in humans, animals, and the environment. This review can be used to define research gaps, improve surveillance, and inform the development of prevention measures considering the One Health holistic approach formulated by the World Health Organization (WHO).

## 2. Materials and Methods

### 2.1. Country of Study

**Figure 1:**
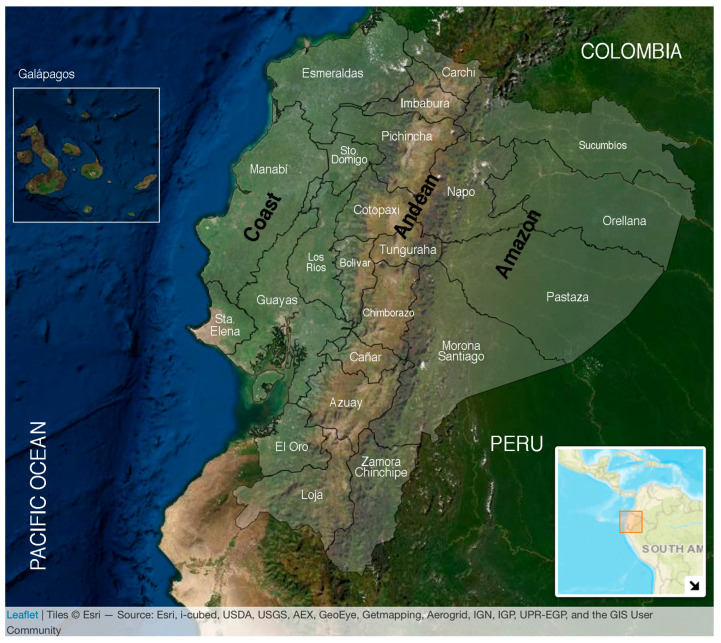
Map of Ecuador. The continental area includes a total of 276,841 km^2^ and is divided into three ecoregions by the Andean Mountains: the Coast in the west, bordering the Pacific Ocean, the Andes in the middle, and the Amazon in the east bordering both Peru and Colombia. Ecuador is divided administratively into the 24 provinces signaled in white words. The Coast encompasses seven (Esmeraldas, Manabí, Santo Domingo de los Tsáchilas, Los Ríos, Guayas, Santa Elena, and El Oro); the Andes encompasses 10 provinces (Carchi, Pichincha, Tungurahua, Chimborazo, Cañar, Azuay, Loja, Imbabura, Bolívar, and Cotopaxi); and the Amazon encompasses six provinces (Sucumbíos, Napo, Orellana, Pastaza, Morona Santiago, and Zamora Chinchipe). The Galápagos Islands are in the Pacific Ocean at ~1000 km from continental Ecuador with a warm climate. The population of each ecoregion was estimated in 2020 as follows: 8,631,859 for the Coast, 7,847,136 for the Andes, 956,600 for the Amazon, and 33,042 for the Galapagos; giving a total population of 17,468,637. Up to 36% of the entire population lives under rural conditions [29]. Tropical and subtropical climates cover approximately 64% of Ecuador’s landmass.

Ecuador is crossed by the equator and is bisected north–south by the Andean Mountain range, the latter dividing the country into three geoclimatic regions: the Pacific Coast and the Amazon with subtropical and tropical rain and dry forests plus the temperate highland Andes, which includes inter-Andean valleys with warm climate. The territory also encompasses a fourth region, the Galápagos Islands (Figure 1).

### 2.2. The Literature Search and Ethics

We surveyed the published literature related to leptospirosis in Ecuador from local, regional, and international journals using PubMed, Scientific Electronic Library (Scielo), Latin American and Caribbean Health Sciences (LILACS), Sistema Regional de Información en Línea para Revistas Científicas de América Latina, El Caribe, España y Portugal (LATINDEX), and Google Scholar. We screened for the following combination of individual terms in any given order: “leptospirosis”, “*Leptospira*”, “febrile illness”, “Amazon”, “Andes”, “Pacific Coast”, “Galapagos”, “Ecuador”, “livestock”, “seroprevalence”, “MAT”, “ELISA”, and “Epidemiology” with no restriction on language or publication date. Nonindexed local journals, bulletins, local meetings, abstracts, theses, and clinical cases were included in our results and discussion. We reviewed the published literature with anonymized data and thus the study does not require any bioethical approval.

## 3. Results

### 3.1. The Published Literature

**Figure 2:**
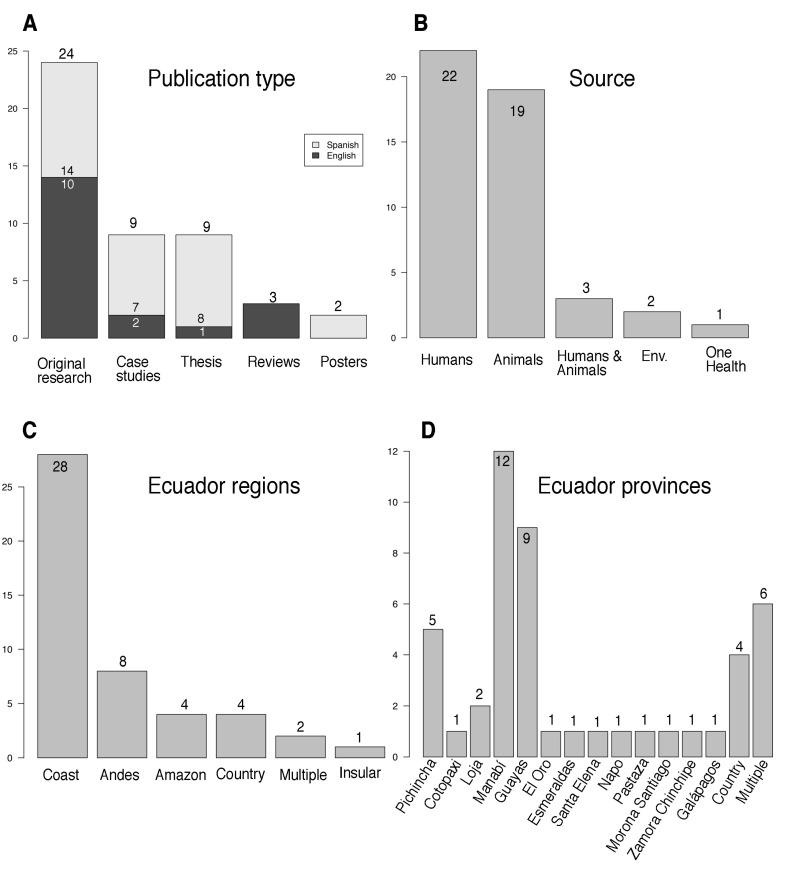
Statistics of the 47 publications on leptospirosis in Ecuador. In (**A**) publication types, in English (black bars) or in Spanish (grey bars); in (**B**) sources of the samples analyzed; in (**C**) by regions; in (**D**) by provinces. “One Health” refers to studies analyzing different *Leptospira* matrices at the same time. “Multiple” refers to studies performed in different regions and provinces at the same time. “Country” refers to studies throughout Ecuador. “Case studies” include both case reports and case series. See Table 1 for details.

We identified 47 publications, 22 (47%) on human infections, 19 (40%) on animals, and two (4.3%) on the environment; three of these articles covered more than one of these areas, while one covered all three topics (i.e., One Health; Figure 2). Thirty-five (75%) research papers were published in the last 11 years (Figure S1); 28 (60%), were performed in the Coastal region; and 24 (51%) appeared in international journals, with 27 (57%) being in Spanish language (Figure 2). Twenty-four were original research (14 in English and 10 in Spanish); three were reviews of national data in English; and two were posters in Spanish. Nine nonindexed research articles (i.e., theses; Figure 2) were published, one in English and eight in Spanish; of those, two studies involved humans; six involved animals; and one—the One Health study—involved humans, animals, and the environment. Table 1 categorizes these references according to the year of publication, summarizes the details included on leptospirosis in humans, animals, and the environment between 1919 and 2022 (103 years), and describe the number of cases, the diagnostic methods, and the serovars identified.

### 3.2. Human Studies

Nine publications depict human cases and/or series reports (seven in Spanish and two in English), eight from the Coast, and one from the Andes (Table 1). Of the studies analyzing only humans, a total of 7342 cases were registered, from which 4557 were positive (62.1%) including two foreign travelers returning from Ecuador [22,24,26,31,32,33,36,37,38,44,45,48,52,55]. In a study of 464 febrile subjects from three communities of the Coastal provinces (Esmeraldas, Manabí, and Guayas), DNA was present in 64% of 210, 25% of 100, and 21% of 154 samples from rural, semiurban, and urban sites, respectively [17]. Five samples from febrile patients tested by the real-time polymerase-chain reaction (qPCR) in Portoviejo-Manabí resulted negative when evaluated during the rainy season [37]. A study of patients from two hospitals in the Amazon region with acute undifferentiated febrile illnesses detected leptospirosis specific-IgM antibodies seroconversion in 14.7% of 227 patients tested [34], while in a cross-sectional seroepidemiology survey in two indigenous Shuar communities in the Amazon-Morona Santiago province, 50% of 216 were positive for IgG antibodies [55]. A further study performed at rural health centers in the Coastal-Manabí detected *Leptospira* spp. DNA in the urine or blood samples in 14.7% of 680 individuals presenting with undifferentiated febrile illness [41].

Misdiagnosis of leptospirosis was reported from 135 febrile-patient samples, while 60% of the patients clinically diagnosed as dengue had antibodies against only *Leptospira*, with 25% of the patients diagnosed as leptospirosis exhibiting antibodies to dengue virus. In the same hospital, clinical records indicated that 72.8% of the patients clinically diagnosed as dengue fever had antibodies to *Leptospira* and not to dengue virus [26]. In another study, two out of seven children diagnosed with hepatitis [25] and two incarcerated adults were initially misdiagnosed with dengue fever or a urinary-tract infection [36].

A review of data from the Integrated Epidemiological Surveillance System (SIVE-ALERTA) of the MoH reported that cases increased from 155 in 2003 to 1279 in 2012 [39], whereas a more recent review reported a decline in incidence between 2013 and 2018 from 3.3 to 0.8 cases per 100,000 population [57]. A retrospective thesis collecting data from the Ecuadorian National Institutes of Public Health and Research (INSPI) recorded 5390 suspected human cases from the Coastal provinces with 1371 (25.4%) positives and indicated an increase in cases from 56 in 2005 to 645 in 2012 [38]. A population-based nationwide study on confirmed hospital-discharged leptospirosis cases from 2000 to 2020 within a publicly accessible National Database recorded 2584 hospitalizations over all three Ecuadorian continental regions (excluding the Galápagos), manifesting an annual incidence of 0.27 to 2.45 cases per 100,000 inhabitants and with 79 fatalities (3.06%) being recorded [12]. No studies on the clinical features, the appropriate treatment, or *Leptospira* spp. sensitivity and/or resistance were found.

### 3.3. Animal Studies

A total of 19 studies investigated 6314 nonhuman animals from which 2105 (33.3%) were found infected with *Leptospira* spp. The studies were performed mainly in the Coast and the Andes, with one study in the Amazon and one in the Galápagos Islands (Table 1, Figure 2). The animals analyzed were composed of (a) domestic ones and livestock: dogs, cattle, horses, sheep, pigs, rabbits, and guinea pigs; (b) wild animals: opossums, sea lions, rats, the South-American coati *Nasua nasua*, the western-mountain coati *Nasuella olivacea*, the northern tiger cat *Leopardus tigrinus*, and two primates (the common woolly monkey, *Lagothrix lagotrichia*, and the Ecuadorian capuchin, *Cebus aequatorialis)* [10,13,14,15,17,23,25,30,37,41,47,49,50,51,53,56,58,59,60].

On the Coastal region, domestic animals in rural areas of Manabí province manifested high positivity rates for pathogenic and intermediate *Leptospira* spp. in urine samples collected from 165 (35.8%) cattle, 128 (21.1%) pigs, and 101 (3.0%) rats [37,41,46]. Of the 90 animal samples collected from Portoviejo-Manabí during the 2009 dry season, 65 (72%) were PCR-positive for *Leptospira* spp.: 21/30 (70%) among dogs, 18/57 (32%) among pigs, and 20/27 (74%) among cattle along with six rat-kidney samples [17]. Another study in rural Manabí employing the MAT for diagnosis revealed positivity rates of 20.6% from 165 pigs raised in backyards compared to 16.5% from 280 pigs raised commercially [59]. In a recent study from an animal refuge in the coastal city of Guayaquil, all 23 domestic and six wild animals surveyed were seropositive for *Leptospira* spp. infection, likewise based on the MAT [15]. Two publications using MAT reported leptospiral infections in cattle-serum over different cantons of the Manabí province as follows: the first reported seropositivity in 57.4% of the 854 samples examined as well as in 97% the herds [10], while the second, in a survey of 749 bovines from 55 herds, found a respective 56.2% and 98.2% positivity, with the most prevalent serovars being Pomona (28.6%) and Icterohaemorrhagiae (22.3%) [60].

In the Andes region, the surveys were concentrated in the Pichincha province [13,23,30,51,53] along with two in Loja [43,61] and one in Cotopaxi provinces [49]. All these studies determined the presence of *Leptospira* spp. infection by MAT, although at least one in Pichincha province used culture in the EMJH medium as well as used a hamster as an animal model for detecting *L. interrogans* in the urine of one of 160 cows examined [13]. The study performed in the southern Andean province of Loja revealed a positivity of 74.8% in a sample of 600 cattle [43]. A second study in Loja city exploring the presence of *Leptospira* spp. In 100 dogs treated at a veterinary hospital found 29% positives [61]. In Cotopaxi, the infection rates of 27.6% (range 12.1–52%), 45.2%, 9%, and 9% were observed in cattle, pigs, dogs, and sheep, respectively [49]. The application of PCR for diagnosis in cattle urine from 547 animals from the Andean Pichincha and the Coastal Santo Domingo de los Tsáchilas provinces detected a prevalence of 13.5% [14].

In the Amazon region, the only study identified was performed in the southeastern province of Zamora Chinchipe; there, cattle from 67 herds were screened by MAT with a total of 26 (12.2%) out of 213 samples proved to be positive [56]. Meanwhile, in the Galápagos Islands, the single PCR study performed on tissue samples from dead sea lions (*Zalophus wollebaeki*) detected DNA for pathogenic *Leptospira* in five out of seven samples collected in 2010 [47].

### 3.4. Environment

Two studies examined the presence of the *Leptospira* in environmental samples (Table 1, Figure 2). The first analyzed the ability of *Leptospira* spp. to survive in rivers within the tropical Coast and the Amazon. Gram-negative *Sphingomonas* spp.—but not *Flavobacterium* spp. or *Delftia* spp.—in in vitro cocultivation supported the survival of the saprophytic *L. biflexa* and an ambiguous species, *L. meyeri*, for up to a year of follow-up in distilled water [35]. The second study evaluated the presence of *Leptospira* spp. in a gradient of water and soil in two rivers from the Coastal-Manabí province. They found a higher prevalence of *Leptospira* spp. in soil than in water, suggesting that dilution of the bacteria could be a major consideration in addressing the causality of infection from water sources. The DNA of the positive samples, however, did not enable a species identification [62]. A third study, likewise performed in Manabí, analyzed samples from humans, animals, and environmental river water (i.e., the One Health approach): the authors used conventional PCR and qPCR and identified *L. kirschneri* in one out of four natural water sources [37].

### 3.5. Leptospira Diversity

All three major clusters of *Leptospira* (i.e., pathogenic, intermediate, and saprophytic) were reported in humans, animals, or the environment in Ecuador (Table 1). Moreover, we recorded the circulation of at least 29 serovars over the three Ecuadorian ecoregions (Tables S1–S3) [10,13,14,15,17,24,25,26,32,37,38,40,41,42,45,49,50,52,53,56,58,60]. A study of animals and febrile patients in rural areas of the Coastal-Manabí province involved molecular-genotyping methods to identify six *Leptospira*—*L. borgpetersenii*, *L. kirschnerii*, *L. santarosai*, *L. interrogans*, *L. noguchii*, and the intermediate species *L. licerasiae* and *L. wolffii* [41]. In another study over the Coast including Esmeraldas, Portoviejo, and Guayaquil cities, leptospiral DNA amplified from febrile patients corresponded to the pathogenic *L. noguchii* (2.7%) and the intermediate-cluster *L. wolffii* (63%) in Esmeraldas and to *L. wolffii* and *L. inadai* (both in the intermediate cluster) in Portoviejo. The analysis of sera from 154 patients of Guayaquil city identified intermediate-cluster *L. wolffii* in 18% and pathogenic *Leptospira* spp. (*L. borgpetersenii* and *L. kirschneri* and/or *L. interrogans)* in 2.5% of samples. *Leptospira kirschneri* and *L. interrogans* could not be differentiated through analysis of DNA isolated from one of the samples studied [17].

From the positive animals identified in Manabí-Portoviejo canton, 19 amplicons were sequenced: three from dogs, three from pigs, seven from cattle, and six from rats. BLAST DNA-sequencing analysis indicated that 14 (74%) had 100% sequence identity to *L. inadai*, whereas amplicons from five animals (three cows, one pig, and one rat) had 100% identity to *L. borgpetersenii* [17]. *Leptospira santarosai* was identified in the urine of human cases from Portoviejo-Manabí province, and *L. kirschneri* was identified from a natural water source. Furthermore, in rat-kidney samples, PCR and DNA sequencing enabled the identification of *L. borgpetersenii* (four samples), *L. noguchii* (two samples), and *L. wolffii* (one sample). Three *Leptospira* spp. were identified in samples of cattle and pigs: *L. borgpetersenii*, *L. interrogans*, and *L. wolffii* (pathogenic cluster). The finding of six different species—*L. borgpetersenii*, *L. wolffii*, *L. santarosai*, *L. kirschneri*, *L. noguchii*, and *L. interrogans*—demonstrated the circulation of multiple species of the pathogenic cluster in Portoviejo canton; of the species found, five were pathogenic and one (*L. wolffii*) was classified as intermediate or of indeterminate pathogenicity [37].

In three studies from the Coast, in one performed at an animal rescue center in Guayaquil city of Guayas province, all domestic and wild animals were seropositive for *Leptospira* spp., with *L. interrogans* serovars Canicola, Hardjo, and Icterohaemorrhagiae being the most frequently identified [15]. In a study on pigs from Manabí province, the most common serovars were Australis and Icterohaemorrhagiae 14.3% (40/280) and Bataviae 13.2% (37/280) [59]. In a more recent study on cattle in the same Manabí province, the most prevalent serovars were Pomona (28.6%) and Icterohaemorrhagiae (22.3%) strains [60].

Three whole-genome *Leptospira* sequences—one obtained from human blood and two from cattle urine collected in Manabí in 2014—enabled the identification of *L. santarosai* in human and *L. interrogans* in cattle samples [42].

Studies on cattle from Pichincha and Santo Domingo de los Tsáchilas provinces have identified *L. borgpetersenii* and *L. inadai* as etiologic species, both analyzed by PCR in urine samples [14]. Cattle examined in the Amazon-Zamora Chinchipe province detected four serovars: Australis, Bataviae, Canicola, and Sejroe [56]. A study examining humans from the Coastal-Guayas province characterized the serovars Autumnalis, Icterohaemorrhagiae, Panama, Patoc, Pomona, and Tarassovi through ELISA and MAT and made the differential diagnosis between leptospirosis and dengue among febrile patients [26].

### 3.6. Laboratory Diagnosis

As to the individual diagnostic methods for *Leptospira* spp. infections, 16 (34%) studies used MAT. Together with ELISA-IgM, this test is the one recommended by the Ecuadorian MoH and is performed for confirming clinical diagnoses for both humans and animals [6,63]. MAT is available at the National Reference Laboratories INSPI (Instituto Nacional de Investigación en Salud Pública; Table 1) for human cases and at laboratories of the Animal Diagnostics Directorate of the Ecuadorian Agency for Agriculture Quality Assurance (AGROCALIDAD, in Spanish) for animal diagnosis. Six studies (15%) without information about the diagnostic method included three reviews, one thesis, and two original research articles [18,39,44,46,54,57]. Fourteen studies used the combination of at least two diagnostic methods, among which MAT and ELISA plus PCR and/or qPCR along with DNA sequencing were the most common technical approaches (Table 1).

### 3.7. Risk Factors for Human and Animal Leptospirosis

In a cross-sectional seroepidemiological survey in two indigenous Shuar communities in the Amazon-Morona Santiago province, data collected by questionnaire on the sociodemographic conditions and the knowledge of infectious diseases including leptospirosis found that the prevalence of pathogens varied by age but not significantly by gender, temporal migration, illiteracy, perceived morbidity, receipt of conditional cash transfers, water-boiling practices, poor housing conditions, or anthropometric status [55]. In another survey involving knowledge, attitudes, and practices (KAP) among cattle workers with a low level of schooling from an endemic region of the Manabí province, 63% reported knowledge of the disease, a figure that was lower than that of a population with a secondary level of scholarship. Women had less knowledge than men. Persons engaged in transporting animals had lower levels of knowledge than farmers, but veterinarians had a higher level. In general, knowledge was deficient on the routes of transmission to animals and humans, the sources of infection, and the measures of prevention [54]. Ruano et al. (2020) found that only cattle age was a risk factor associated with leptospirosis [60].

A study in the Coast revealed that the prevalence of human leptospirosis increased in the middle of the rainy season—only to rebound in May, June, July, and August—which as a period corresponds to the time of stagnating water and the formation of ponds; toward the end of the year, the prevalence decreased but remained constant [38]. In a study addressing the number of human hospitalizations for leptospirosis nationwide during a twenty-one-year period, the incidence of leptospirosis increased in the Coast between January and May. By contrast, in the Andes the incidence of hospitalized cases remained relatively constant throughout the year, whereas in the Amazon the value both increased and decreased during the year but did so without a clear pattern [12].

## 4. Discussion

The publications scrutinized in the present review confirmed that leptospirosis was and still is a zoonotic and endemic disease of Ecuadorian nationwide distribution affecting humans, livestock, and both domestic and wild animals with *Leptospira* spp. that can be readily isolated from the environment. All four ecological regions (Coast, Andes, Amazon, and the Galápagos Islands) reported animal infections, mostly in the two tropical ecoregions. Outbreaks of human and animal infections occurred in urban, suburban, and rural populations, with the majority occurring in rural areas, particularly during the rainy season [12,16,17,18,34,38]. Moreover, according to three studies analyzing the national dataset, clinical cases have been increasing during the last two decades, especially in the Coastal provinces [12,38,39]. The high positivity rate of leptospiral infections in both humans and animals especially in rural communities indicated a high level of transmission. All these findings taken together point to the fact that leptospirosis in Ecuador is an emerging public health problem for local populations as well as for international visitors [33,64].

The infections recorded in domestic and wild animals in rural and urban areas nationwide indicated that leptospirosis is endemic in Ecuador and thus represents a potential risk for human infections and environmental contamination [10,13,14,15,17,23,30,37,47,49,50,51,53,58,59,60]. Infected sea lions with pathogenic leptospires were found in the Galápagos Islands, where contact between local inhabitants or tourists and domestic and wild fauna is frequent [47]. Nonetheless, the impact of these infections is unclear. No human cases have been recorded yet in the Galápagos but might be found by implementing active research on local populations or on other animals living in the Islands. None of the studies addressed clinical manifestations, morbidity, and mortality in animals, a critical gap that needs urgent attention since leptospirosis can cause enormous veterinary economic losses [65].

Although leptospirosis is considered endemic and a public health concern by the Ecuadorian MoH, an operational national control program does not exist, nor have clinical-practice guidelines been established, in contrast to the situation for other infectious diseases in the country, e.g., malaria, leishmaniasis, dengue, and tuberculosis (https://www.salud.gob.ec/guias-de-practica-clinica-2019, accessed on 24 August 2022). Nevertheless, human leptospirosis is of mandatory notification to the National Directorate of Epidemiological Surveillance, and accordingly, the MoH report the cases weekly in the Statistical Reports section of Ecuador Epidemiological Gazette, SIVE-ALERTA [6]. The cases reported, however, have been diagnosed clinically and usually have not been confirmed by paired serological and/or bacteriological tests, thus being the reason underlying the discrepancy between the leptospirosis patterns described from SIVE-ALERTA data [57] and the figures obtained from confirmed hospitalized cases recorded by INEC [12]. A shortcoming of great concern is that Ecuador lacks a committee to review leptospirosis cases in order to reach a definitive conclusion. Furthermore, the country lacks an ongoing team to take action, develop preventive measures, or assume control in situations involving possible outbreaks or during the floodings that constitute known risk factors for leptospirosis.

In Ecuador, MAT is considered the diagnostic method of choice for laboratory diagnosis and is performed on suspicious samples that are positive for the ELISA test, following PAHO guidelines. ELISA is performed in the province capitals of the Coastal and Amazon regions [38]. MAT measures seroconversion or the increase in *Leptospira* antibody titers. Nevertheless, few of those sera are tested in paired samples [38]. INSPI is the only reference center nationwide for diagnosis of *Leptospira* infection by means of MAT. The diagnosis is available for 27 (ATCC) serovars, though all are often unavailable (Table S1). Molecular-genetic typing is not performed although that technique is strongly recommended [4].

For animals, leptospirosis is included in the list of terrestrial animal diseases under surveillance, with the regulation agency AGROCALIDAD conducting diagnoses by MAT. The detection capability for animals, however, was limited to 12 serovars. For this reason, Ecuador lacks updated and publicly available data on the incidence and prevalence of leptospiral serovars or on the geographic distribution of animal infections. Therefore, up-to-date data from INSPI and AGROCALIDAD are urgently needed. Ecuador lacks an official national program to react to leptospirosis outbreaks in animals because of insufficient field personnel to obtain samples. No compulsory vaccination program exists for any species of animal. Vaccines are available in the private sector and are implemented only at the request of those who are interested and can afford them. Since vaccination in cattle has evidenced a reduction in *Leptospira* shedding [8], animal vaccination in dairy farms is likely to be an effective control measure.

Because only 47 publications during the entire 103-year period were identified here, we can state that *Leptospira* and leptospirosis are neglected areas of study in Ecuador. In the unique ‘One-Health’ study performed in the Coast region with limited samples, the few positive results from river water and human sera examined prevented the demonstration of correlations of *Leptospira* species among the different samples involving the environment, animals, and humans [37]. Future studies are highly recommended in other localities with large numbers of samples to understand the dynamics of leptospirosis in relation to the soil and freshwater. Among these 47 papers, the majority were focused on humans, with some reports being clinical cases, predominantly performed in the Coast, with only three reviews analyzing nationwide data from the MoH [12,38,39,57]. Most of the studies (81%) were published in the last 2 decades. Only 18 were found in PubMed, with most of the research data remaining in the gray literature published in local journals and in Spanish. Most of the research was financed and performed by private institutions. More than half (27/47) of the literature was written in Spanish and published in national journals or were theses that are unregistered in international platforms. Furthermore, not all publications were correlated with the burden that the disease represents in Ecuador, even though that information was known as early as 12 decades ago [20]; in addition, most of the studies were retrospective, with none covering all four ecoregions at the same time.

Moreover, not a single study has been either performed or published involving aspects of clinical manifestations, routes of transmission, modes of treatment, and bacterial antibiotic sensitivity or resistance. Thus, health and veterinary personnel are uninformed and are unaware of the challenges to medical practice involving either leptospirosis case management or the potentiality for epidemics in Ecuador. Therefore, several human cases have been and are still being mistakenly diagnosed with other febrile illnesses [17,20,23,24,25,26,34,36]. Thus, we evidenced clinical overdiagnosis; i.e., in samples tested at the INSPI for a confirmatory laboratory diagnosis, only 25.4% of the clinical samples were verified as being positive for leptospirosis [38]. We therefore encourage health authorities to educate healthcare personnel and local populations about the transmission routes of *Leptospira*, the several clinical features, and the management of the disease. Identification of the etiologic agent is critical for disease treatment in Ecuadorian tropical regions where other febrile illnesses—such as dengue, malaria, brucellosis, Chagas disease, Zika, chikungunya, Q fever, and more recently COVID-19—are overlapping [6,11,26,66]. Simple and rapid diagnostic methods for common febrile tropical infections including leptospirosis should be readily available, as well as the recommended antibiotics for a specific clinical situation. The epidemiology of leptospirosis in remote tropical rural areas of the Coastal and the Amazonian provinces as well as the Galápagos Islands has been poorly studied, with wide gaps in the knowledge, even though up to 36% of the Ecuadorians live in rural areas [29].

The Pacific coastal region has been the most widely studied, with 28 (60%) investigations in humans, animals, and the environment; particularly in the rural zones of the Manabí province with 12/47 (26%) studies, where several outbreaks of leptospirosis have been confirmed by the INSPI [38,67], thus demonstrating a lack of research in the other three ecoregions. Accordingly, the performance of epidemiological studies in these areas—from a One Health perspective—would be highly advisable to fully characterize the epidemiology of leptospirosis within the entire country.

Since four studies evidenced that leptospirosis was a major cause of febrile illness in the tropics [17,26,34,55], we suggest performing MAT seroconversion on paired serum samples in patients presenting with fever from all tropical areas of Ecuador, especially from clusters in cantons of the Manabí-Coastal and Zamora Chinchipe-Amazon regions, both were identified as being at high risk of *Leptospira* infection [6,12,17,57]. Leptospiral DNA in Ecuadorian febrile patients increased following an ascending gradient from urban to suburban to rural areas [17]. Because of the high incidence in rural inhabitants engaging in outdoor activities such as cattle raising and agriculture, e.g., the rice crops in the Coast [38], leptospirosis should be recognized as an occupational disease. In addition, research should be performed on workers of slaughterhouses and on people within the dairy, sheep, beef, and pork industries with the purpose of implementing effective control measures such as the use of personnel protection in farms and workplaces to avoid infections among these potential high-risk groups.

Studies on cattle reported varying rates of *Leptospira* spp. infection, ranging from 35.8% to 75%; at the herd level, the prevalence reaches up to 97% in the Manabí province [10]. In addition, the high percentage of positive urine from slaughterhouse animals [14,56] indicated that cattle were major natural reservoirs, carriers, and dispersers of pathogenic *Leptospira*. Since the positivity rate in rats was lower than that in pigs and cattle, we assume that swine and bovines play a more fundamental role than rats in the transmission within the study area. What is striking is that positive but ostensibly healthy cattle were maintained for considerable times in farms, thus posing a risk to owners and noninfected animals [54]. Therefore, positive, though seemingly healthy livestock might be principal sources of environmental contamination and transmission in agricultural communities. Although *Leptospira* are less concentrated in livestock urine than in rat urine, the greater volume in cattle might result in a higher extent of bacterial shedding [46]. Thus, nationwide livestock investigations for leptospiral infection should be a crucial endeavor within a national surveillance and control strategy. Different reservoir species would influence the epidemiology of *Leptospira* spp. and affect the development of effective disease prevention and control strategies.

In Ecuadorian large tropical cities, rats are abundant [41,50,68] and potentially act as the main propagators of the *Leptospira* spp. infecting humans and new animal reservoirs. The high percentage of positive rat-kidney samples [17] urge the need to monitor rodent populations and freshwater during the rainy season since those conditions potentiate the endemicity. Thus, the role of rats as principal propagators of pathogenic and nonpathogenic *Leptospira* still remains to be a challenge in the country. Studies and interventions in nonhuman animals comprise an extensive field for research. In Ecuador, the interest in studying leptospirosis in wildlife is moderate, and because of bureaucracy and logistics, research has focused mainly on livestock. We would stress, however, the need for more research on wildlife at the local and regional scale. Identification of the prevailing genotypes and their animal reservoirs is essential to understand the epidemiologic characteristic of different niches in order to advise specific measures of infection prevention and control [4,15,69].

Three studies have been performed in Ecuador with environmental samples, one labeled here as a ‘One-Health’ study, published as a thesis within the gray literature [35,37,62]. Leptospires can survive for months in humid, warm environments and in abundant surface water and soils before being transmitted to mammalian hosts [2]. Leptospires were isolated from a river of the Amazon, and that their cocultivation with *Sphingomonas* spp. enabled survival of *L. biflexa* and *L. meyeri* for up to a year in distilled water [35] is an informative epidemiologic revelation regarding *Leptospira* survival. We encourage studies on water and/or soil in tropical environments but also within the temperate regions or the inter-Andean valleys, where leptospirosis has also been diagnosed [12]. The isolation and identification of environmental leptospires from different settlements will prevent potential future infections/outbreaks. Inhabitants of rural villages of the Coast and Amazon usually walk barefoot and lack potable water or basic sanitation; moreover, they routinely drink and use water from rivers or waterholes or stored water, which are known to maintain viable infective leptospires [55,59]. As leptospirosis is considered an environmental water-based disease, we suggest further studies in streams, rivers, lakes, lagoons, and ponds and on shores applying molecular-genetic techniques (PCR and DNA sequencing) to characterize pathogenic, intermediate, and saprophytic clusters or the taxonomic groupings in accordance with the newly proposed classification into the four subclades P1, P2, S1, and S2 [4]. These analysis should be conducted in both rainy and dry seasons since *Leptospira* could persist for extensive periods in nutrient-poor environments [35]. For example, in the Coastal region, human cases increased just in the middle of a rainy season but rebounded during dry months [38], a pattern that was also recorded upon consideration of the number of leptospirosis hospitalizations [12].

A study comparing human and animal samples collected in the Coast from local health centers and local slaughterhouses, respectively, found a genotypic match between both populations but a lack of evidence for contact between infected individuals [17]. These findings underscore the need to conduct longitudinal surveys of leptospiral populations and moreover warrant further research on the effect of *Leptospira* spp. on the disease severity observed in veterinary and human infections.

The three classical clusters of *Leptospira*—pathogenic, intermediate, and saprophytic—have been described over the four geoclimatic regions of Ecuador [10,17,37,47,67]. In three Coastal provinces, the intermediate cluster was far more prevalent (96%) than the pathogenic (4%) in humans, whereas the respective prevalence of those same clusters in their animals were 49% and 51% [17]. Apparently, the intermediate group was causing a substantial amount of undifferentiated fever throughout coastal Ecuador. Six *Leptospira*—*L. borgpetersenii*, *L. wolffii*, *L. santarosai*, *L. kirschneri*, *L. noguchii*, and *L. interrogans*—were described in the Portoviejo canton-Manabí province, an area that evidenced a high diversity of pathogenic and intermediate clusters, as reflected in the high proportion of infected residents in the region [67]. Burgos-Macias et al. (2019), investigating cattle in the same province, reported the presence of eight serovars—i.e., Canicola, Hardjo, Pomona, Icterohaemorrhagiae, Grippotyphosa, Wolffi, Bratislava, and Copenhageni [10]. The identification of *Leptospira* at the species and serovar levels is essential since differences have been associated with disease severity and therefore can be epidemiologically and clinically informative. Unfortunately, the laboratories and human expertise are restricted to only a few reference facilities in the country. The implementation of diagnoses and molecular-genetic identifications should be encouraged in at least the reference laboratories of INSPI, where the molecular-genetic diagnosis of other infectious diseases is already a standard procedure.

In tropical Ecuador, a higher frequency of floodings occurs in the Coast, with Guayas being the province most greatly affected, followed by Manabí, Los Ríos, Esmeraldas, and El Oro [70]. We need to note that rainfall is longer and warmer in the Amazon region, but the population and density per m^2^ is lower than in the other two continental regions [71,72]. Ecuador, because of its geography, geology, oceanography, climate, and demography, is a country vulnerable to the effects of climate change [73]. In recent years, the country has registered sustained increases in temperature, and the annual amount of precipitation has increased throughout the Coast. The average annual precipitation has increased 33% in the Coast and 8% in the Andes [71], imposing the risk of increased cases of leptospirosis because of the pathogen’s water-based transmission route [2,74]. The official human–leptospirosis data collected along with nationwide reports indicated an association with rainy months [12,38], similar to what has been found globally [2]. One study reported more cases in the Coast during the rainy period, particularly between the months of February through June [41], thus suggesting that climatic conditions drive the increased numbers of cases observed in tropical regions [71]. In addition, Calvopiña et al. (2022) observed an increase in hospitalized cases in the Coast but not the Amazon or the Andes between the months of January and May [12]. Therefore, rainfall and stagnant water, contaminated environments, and lack of sanitation constitute some of the main risk factors responsible for the occurrence of leptospirosis in the country.

Sixteen (34%) studies used MAT, which is only available for human samples at the INSPI in only the three main cities of the country (Guayaquil, Quito, and Cuenca). AGROCALIDAD is also a governmental institution that applies this test but focuses on animal populations with laboratories in the Coast and Andes but not in the Amazon. MAT performed by the INSPI identified 26 serovars, including the pathogenic but not all the intermediate cluster, e.g., *L. fainei* and *L. licerasiae*, that have been identified in high numbers (96%) in febrile patients of the Coast [17]. Those serovars were causally implicated in mild and chronic infections [3]. The application of point-of-care diagnosis to identify cases of acute leptospirosis is urgently needed.

Only seven (15%) studies used DNA-based techniques (PCR or DNA sequencing), which are recommended for characterizing and identifying *Leptospira* spp. In general, molecular-genetic techniques are more sensitive for diagnosing active infections than culture, ELISA, or MAT, as demonstrated in several studies in Ecuador [14,17,35,37,40,41,47]. In this country, molecular tests are not available for diagnostic purposes of leptospirosis and are performed only at the private sector and for research purposes. Although in recent times several studies have applied molecular methods in Ecuador, certain results suggested a limited degree of specificity of the tests [37], and therefore the approach needs to be standardized throughout the country.

Finally, we encourage that the official system of epidemiological reporting should be improved because a disagreement between reports of local and national authorities was notable: for example, during 2010–2012, in Portoviejo-Manabí province, >2000 serologically confirmed cases were reported by local health authorities [17] although the official SIVE-ALERTA surveillance registered 1784 cases [39].

## 5. Conclusions

The epidemiology of leptospirosis has not been extensively studied in Ecuador. This problem must be addressed from a One Health perspective involving multidisciplinary and multisectoral approaches and including both animal and ecologic matrices. Leptospirosis should be considered as a reemerging zoonosis, a heretofore forgotten disease, and a current public health hazard in Ecuador. Gaps exist in our technical and scientific knowledge that hinder epidemiological surveillance, clinical management, prevention, and control of this pathology. Furthermore, the lack of a clinical guide or a standard protocol for control strategies from the Ecuadorian health authorities for assessing leptospirosis in the country reinforces the status of leptospirosis as a neglected zoonotic disease. Even with the recent progress made by researchers and government reports, major hiatuses in our knowledge still hamper efforts of surveillance and prevention. Policymakers and stakeholders could use the information presented in this review to implement well-focused programs of prevention and health promotion to break the cycle of leptospiral transmission. These approaches include carrying out targeted research, building partnerships between key stakeholders, conducting training at different levels, and encouraging investment. After acknowledging the veracity of this informational shortcoming, the government of Ecuador should launch a national program for the prevention and control of leptospirosis and create an expert committee in the endemic Coast and Amazon regions aiming to reduce the morbidity, mortality, and economic losses associated with *Leptospira* spp. infections.

## Figures and Tables

**Table 1:** Articles on *Leptospira* spp. and leptospirosis published in Ecuador since 1919 until 2021. Studies are alphabetically order considering the first author’s name. Details of specific studies (in bold) can be found at the end of the table.

First Author/ Pub. Year	Article Type	Region/ Province	Language/ Target	Sample Source	Studied Cases	Positive Cases	Diagnostic Method	*Leptospira* spp.	Serovars
1. Noguchi H, 1919 [20]	Original research	Coast/ Guayas	English/ International	Human	172	172	Serology	NA	NA
2. Carbo Noboa JM, 1924 [22]	Case report	Coast/ Guayas	Spanish/ National	Human	1	1	Culture	NA	NA
3. Bravo M & Leon P, 1962 [23]	Original research	Andes/ Pichincha	Spanish/ National	Cattle	1780	216	Serum agglutination *	*interrogans*	Pomona
4. Barrera Sosa O, 1970 [24]	Case report	Coast/ Guayas	Spanish/ National	Human	1	1	Culture, MAT	*interrogans*	Grippotyphosa
5. Dávila A et al., 1979 [27]	Original research	Coast/ Guayas & Los Rios	Spanish/ National	**Multiple animals ø**	117	23	Culture, Microscopy	NA	NA
6. Yépez W et al., 1979 [25]	Original research	Coast/ Guayas & Los Ríos	Spanish/ National	Human	8	3	MAT	*interrogans*	Australis, Cynopteri, Hyos, Wolffi,
7. Chavez J, 1985 [30]	Original research	Coast & Andes/ Guayas & Pichincha	Spanish/ National	Pigs	200	99	MAT	NA	Australis, Autumnalis, Bataviae, Canicola, Castellonis, Copenhageni, Grippotyphosa, Hebdomadis, Pomona, Pyrogenes, Sejroe, Tarassovi, Wolffii
8. Gutierrez EC, 1986 [13]	Thesis	Andes/ Pichincha	Spanish/ National	Cattle	160	1	Culture, Microscopy	*interrogans*	NA
9. Jibaja E et al., 2000 [31]	Case report	Coast/ Esmeraldas	Spanish/ National	Human	1	1	Histophatology	NA	NA
10. Chedraui P et al., 2001 [32]	Case report	Coast/ Guayas	Spanish/ National	Human	1	1	MAT	*interrogans*	Ballum, Icterohaemorrhagiae
11. Gelman S et al., 2002 [33]	Case series	Coast/ Guayas	English/ International	Human	2	2	Culture	NA	NA
12. Manock SR et al., 2009 [34]	Original research	Amazon/ Pastaza	English/ International	Human	533	40	ELISA	NA	NA
13. Baquero-Cardenas MI, 2011 [14]	Thesis	Coast & Andes/ Pichincha & Sto. Dgo. Tsáchilas	English/ National	Cattle	547	73	PCR & DNA sequencing	*borgpetersenii, inadai*	NA
14. Barragán V et al., 2011 [35]	Original research	Amazon/ Napo	English/ International	Environment	NA	NA	Culture, PCR	*biflexa, meyeri, santarosai*	NA
15. Gamboa AA et al., 2013 [26]	Original research	Coast/ Guayas	English/ International	Human	135	18	ELISA, MAT	NA	Autumnalis, Icterohaemorragiae, Panama, Patoc, Pomona, Tarassovi
16. Valarezo-Sevilla D et al., 2014 [36]	Case series	Coast/ Manabí	English/ International	Human	2	2	ELISA	NA	NA
17. Sosa A, 2015 [37]	Thesis	Coast/ Manabí	Spanish/ National	Multiple hosts **	295	19	PCR	*borgpetersenii, interrogans, kirschneri, noguchii, santarosai, wolffii*	NA
18. Chiriboga J et al., 2015 [17]	Original research	Coast/ Multiple †	English/ International	**Multiple hosts ††**	670	254	PCR & DNA sequencing	*borgpetersenii, inadai, kirschneri, wolffii*	NA
19. Mendoza R, 2015 [38]	Thesis	**Coast/** **Multiple º**	Spanish/ National	Human	5443	1371	MAT, ELISA	*santarosai*	Australis, Autumnalis, Babudieri, Babudieri, Bataviae, Borincana, Bratislava, Canicola, Celledoni, Celledoni, Copenhageni, Djasiman, Cynopteri, Hardjo, Icterohaemorrhagiae, Javanica, Panama, Patoc, Pomona, Pyrogenes, Saxkoebing, Sejroe, Shermani, Wolffi
20. Cartelle-Gestal M et al., 2015 [39]	Review	Nationwide/ Nationwide	English/ International	Human	NA	NA	NA	NA	NA
21. Salinas A et al., 2016 [40]	Poster	Coast/ Manabí	Spanish/ National	Human	576	2	RT-PCR	*interrogans, santarosai*	Icterohaemorrhagiae
22. Barragan V et al., 2016 [41]	Original research	Coast/ Manabí	English/ International	**Multiple hosts ºº**	1002	173	RT-PCR & DNA sequencing	*Borgpeterseni, interrogans, kirschnerii, noguchii, santarosai, wolffii*	NA
23. Barragan V et al., 2016 [42]	Original research	Coast/ Manabí	English/ International	Multiple hosts ***	2	2	Culture & DNA sequencing	*interrogans, santarosai*	NA
24. Roman-Cárdenas F & Chávez-Valdivieso R, 2016 [43]	Original research	Andes/ Loja	Spanish/ National	Cattle	600	449	MAT	NA	NA
25. Zambrano P et al., 2017 [44]	Original research	Coast/ Manabí	Spanish/ International	Human	248	248	NA	NA	NA
26. Zambrano-Sanchez G et al., 2017 [45]	Case report	Andes/ Pichincha	Spanish/ National	Human	1	1	ELISA	NA	NA
27. Barragan V et al., 2017 [46]	Review	Coast/ Manabí	English/ International	Cattle	NA	NA	NA	NA	NA
28. Denkinger et al., 2017 [47]	Original research	Galápagos Islands/ Galápagos Islands	English/ International	Sea lions	7	5	PCR & DNA sequencing	NA	NA
29. Chamaidan-Ramon G & Loaiza-Guzman FM, 2018 [48]	Case report	Coast/ El Oro	Spanish/ National	Human	1	1	MAT, ELISA	NA	NA
30. Lascano P et al., 2018 [49]	Original Research	Andes/ Cotopaxi	Spanish/ National	**Multiple animals &&**	252	NA	MAT	*interrogans*	Canicola, Icterohaemorrhagiae, Pomona, Sejroe, Tarassovi
31. Campos J, 2018 [50]	Poster	Coast/ Guayas	Spanish/ National	Rats	30	16	MAT	*interrogans*	Autumnali, Bataviae, Canicola, Celledoni, Copenhageni, Djasiman, Grippotyphosa, Icterohaemorrhagiae, Javanica, Pomona, Sejroe, Saxkoebin, Tarassovi
32. Meneses E, 2018 [18]	Thesis	Nationwide	Spanish/ National	Human	NA	NA	NA	NA	NA
33. Maldonado A et al., 2018 [51]	Thesis	Andes/ Pichincha	Spanish/ National	Dogs	90	5	MAT	NA	Canicola, Grippotyphosa, Icterohaemorrhagiae
34. Torres P & Miño G, 2019 [52]	Case report	Coast/ Guayas	Spanish/ International	Human	1	1	MAT	NA	Shermani
35. Chiriboga C, 2019 [53]	Thesis	Andes/ Pichincha	Spanish/ National	**Multiple animals $$**	198	62	MAT	NA	Bataviae, Bratislava, Canicola, Hardjo, Sejroe
**36. Burgos-Macías DI et al., 2019 ß** **[54]**	Original research	Coast/ Manabí	Spanish/ International	Human	NA	NA	NA	NA	NA
37. Burgos-Macias DI et al., 2019 [10]	Original research	Coast/ Manabí	English/ International	Cattle	854	490	MAT	*interrogans*	Canicola, Hardjo, Pomona, Icterohaemorrhagiae, Gruppotyphosa, Wolffi, Bratislava, Copenhageni
38. Romero-Sandoval N et al., 2019 [55]	Original research	Amazon/ Morona Santiago	English/ International	Human	216	108	ELISA	NA	NA
39. Muyulema EH, 2020 [56]	Thesis	Amazon/ Zamora Chinchipe	Spanish/ National	Cattle	213	26	MAT	*borgpeterseni, interrogans*	Australis, Bataviae, Canicola, Sejroe
40. Núñez-González S, 2020 [57]	Review	Nationwide/ Nationwide	English/ International	Human	NA	NA	NA	NA	NA
41. Orlando SA et al., 2020 [15]	Original research	Coast/ Guayas	English/ International	**Multiple animals ||**	29	29	MAT	*interrogans*	Australis, Autumnalis, Bataviae, Canicola, Copenhageni, Cynopteri, Hardjo, Icterohaemorrhagiae, Grippotyphosa, Pomona, Sejroe, Tarassovi, Wolfii
42. Orlando SA et al., 2020 [58]	Original research	Coast/ Santa Elena	English/ International	Horses	108	108	MAT	*borgpetersenii, interrogans, kirschneri*	Bataviae, Bratislava, Canicola, Grippotyphosa, Sejroe, Tarassovi
43. Zambrano-Gavilanez MP et al., 2020 [59]	Original research	Coast/ Manabí	Spanish/ International	Pigs	280	53	MAT	*interrogans*	Australis, Bataviae, Canicola, Hardjo, Icterohaemorrhagiae, Sejroe, Tarassovi, Wolffi
44. Ruano M et al., 2020 [60]	Original research	Coast/Manabí	Spanish/ International	Cattle	749	421	MAT	*interrogans*	Bratislava, Canicola, Copenageni, Grippotyphosa, Hardjo, Icterohaemorrhagiae, Pomona, Wolffii
45. Pinta D, 2020 [61]	Thesis	Andes/Loja	Spanish/ National	Dogs	100	29	MAT	NA	Autumnalis, Canicola, Hebdomadis, Patoc, Pomona
46. Miller et al., 2021 [62]	Original research	Coast/Manabí	English/ International	Environment	72	11	RT-PCR & DNA sequencing	NA	NA
47. Calvopiña et al., 2022 [12]	Original research	Nationwide	English/ International	Human	NA	2584	MAT, ELISA	NA	NA

Destails of specific publications: * Bravo M & Leon P, 1962 [23]: Serum agglutination using the Stoenner method. ø Dávila A et al., 1979 [27]: Included different rat species = *Rattus rattus*, *R. alexandrinos*, *R. novergicus* and opposums (*Didelphis marsupialis*). The manuscript does not specify the number of each animal category. ** Sosa A, 2015 [37]: Multiple hosts include Humans = 159, Animals: rats = 80, pigs = 30, cattle = 26, Environment = freshwater. † Chiriboga J, et al., 2015 [17]: Multiple provinces include Esmeraldas, Manabí & Guayas. †† Chiriboga J, et al., 2015 [17]: Multiple hosts include: Humans = 464, Animals: Dogs = 30, Pigs= 57, cattle = 54, rats = 66. º Mendoza R, 2015 [38]: Multiple provinces include: Manabí, Esmeraldas, Santo Domingo de los Tsáchilas, Los Ríos, Guayas, Santa Elena, El Oro. ºº Barragan V, et al., 2016, a [41]: Multiple hosts include: Humans = 608; Animals: cattle = 165, pigs = 128, rats = 101. *** Barragan V, et al., 2016, b [42]: Multiple hosts include: Human = 1, Cattle = 1. && Lascano P, et al., 2018 [49]: Publication does not specify the number of categories that belong to either dogs or cattle. Thus, we did not include them in the total sum of cases. $$ Chiriboga C, 2019 [53]: Multiple animals include: Cattle= 77, sheep = 68, dogs = 21, pigs = 32. ß Burgos-Macías DI, et al., 2019 [54]: Publication that evaluates knowledge, attitudes, and practices. || Orlando SA, et al., 2020 [15]: Multiple animals include: Domestic: dogs = 4, horses = 3, cattle = 3, sheep = 3, pigs = 3, rabbits = 3, guinea pig = 1; Wild = lions = 3, *Nasua nasua* = 2, *Nasuella olivacea* = 1, *Leopardus tigrinus* = 1, *Lagothrix lagotrichia* = 1, *Cebus aequatorialies* = 1.

## Data Availability

Not applicable.

## Supplementary Materials

The following supporting information can be downloaded at: https://www.mdpi.com/article/10.3390/tropicalmed8040202/s1, Figure S1: Number of publications about leptospirosis in Ecuador included in this review distributed by decades. Thirty-five (74.5%) articles were published in the last 11 years with most studies developed in the 2011–2020 decade, Table S1: Twenty-seven serovars of *Leptospira* spp. used in the microagglutination test (MAT) at INSPI-Ecuador, Table S2: Species of *Leptospira* identified in Ecuadorian provinces. Studies included those that specifically refer to species independent of the serovar identified. A total of nine species have been identified in this review. Table S3: Serovars of *Leptospira* identified in Ecuadorian provinces. Studies included those that specifically refer to Serovars. Publications lack information on the species identified. A total of 29 serovars have been identified in this review.
