# Supplementary material for: Leptospirosis in Ecuador: Current Status and Future Prospects
Source: Trop Med Infect Dis. 2023 Mar 29;8(4):202. doi: 10.3390/tropicalmed8040202 (PMC10141158; doi:10.3390/tropicalmed8040202)
Supplement: Supplementary file 1 [file tropicalmed-08-00202-s001.zip › tropicalmed-2264801-supplementary.pdf]

Leptospirosis in Ecuador: Current Status and Future Prospects

Manuel Calvopiña, Daniel Romero-Alvarez, Eduardo Vasconez, Gabriela Valverde-Muñoz, Gabriel Trueba, Miguel Angel Garcia-Bereguiain and Solon Alberto Orlando

Supplementary material

Figure S1. Number of publications about leptospirosis in Ecuador included in this review distributed by decades. Thirty-five (74.5%) research papers were published in the last 11 years with most studies performed in the 2011-2020 decade.

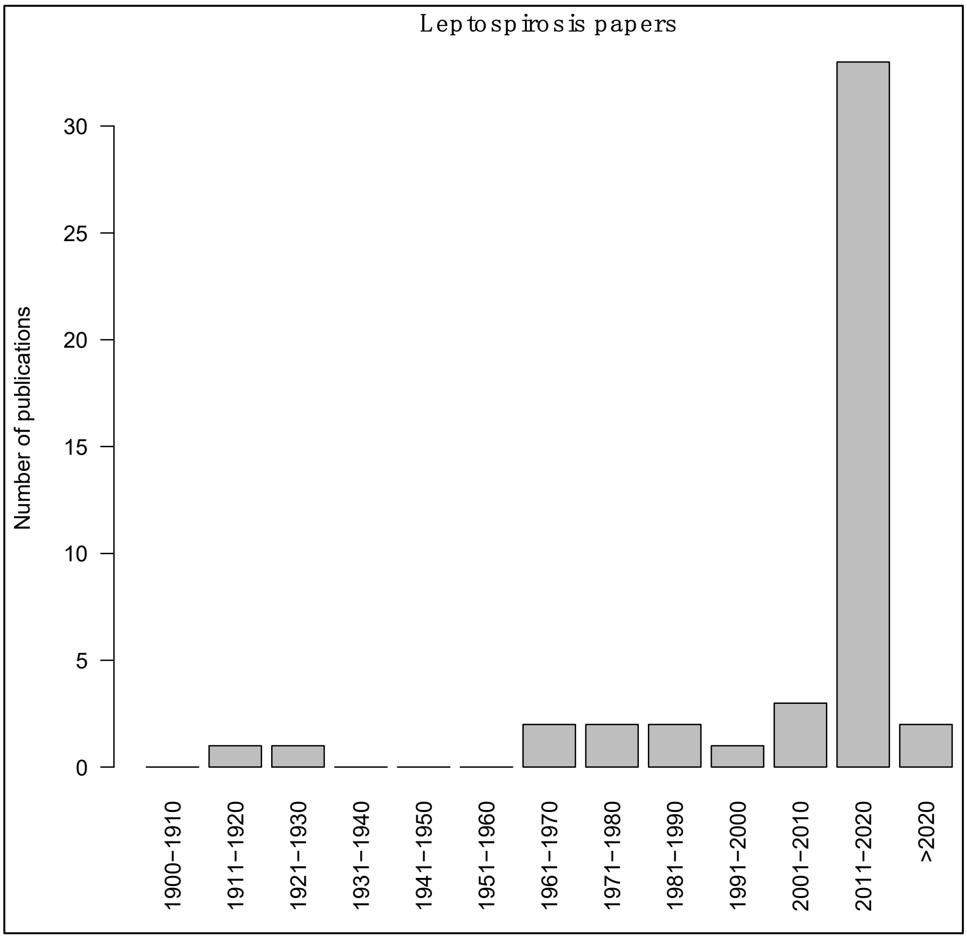

Table S1. Current list of *Leptospira* spp., serogroup, serovars, and strains detected in Ecuador according to the microagglutination test (MAT).

Nº	SPECIES	SEROGROUP	SEROVAR	STRAIN
1	*L. santarosai*	Shermani	Shermani	1342 k
2	*L. interrogans*	Bataviae	Bataviae	Van tienen
3	*L. kirschneri*	Cynopteri	Cynopteri	3522c
4	*L. interrogans*	Hebdomadis	Hebdomadis	Hebdomadis
5	*L. borgpetersenii*	Tasassovi	Tasassovi	Perepelitsin
6	*L. interrogans*	Icterohaemorrhagiae	Icterohaemorrhagiae	Ictero I
7	*L. borgpetersenii*	Sejroe	Saxkoebing	Mus 24
8	*L. interrogans*	Sejroe	Hardjo	Hardjoprajtino
9	*L. interrogans*	Pomona	Pomona	Pomona
10	*L. interrogans*	Sejroe	Wolffi	3705
11	*L. interrogans*	Autumnalis	Autumnalis	Akiyami A
12	*L. interrogans*	Canicola	Canicola	Hond Utrecht IV
13	*L. weilii*	Celledoni	Celledoni	Celledoni
14	*L. kirschneri*	Grippotyphosa	Grippotyphosa	Moska V
15	*L. interrogans*	Pyrogenes	Pyrogenes	Salinem
16	*L. interrogans*	Australis	Bratislava	Jez Bratislava
17	*L. santarosai*	Hebdomadis	Borincana	HS 622
18	*L. noguchii*	Panama	Panama	CZ 214
19	*L. interrogans*	Icterohaemorrhagiae	Copenhageni	M20
20	*L. borgpetersenii*	Ballum	Castellonis	Castellon 3
21	*L. borgpetersenii*	Javanica	Javanica	Veldrat Batavia 46
22	*L. interrogans*	Australis	Australis	Ballico
23	*L. Borgpetersenii*	Sejroe	Sejroe	M 84
24	*L. interrogans*	Bataviae	Bataviae	Swart
25	*L. interrogans*	Djasiman	Djasiman	Djasiman
26	*L. biflexa*	Semaranga	Patoc	Patoc I
27	*L. biflexa*	Andaman	Andamana	CH 11

Table S2. Species of *Leptospira* identified in Ecuadorian provinces. Studies included those that specifically refer to species independent of the Serovar identified. A total of nine species have been identified in this review. *Multiple include Guayas, Los Ríos, Pichincha, Esmeraldas, and Santo Domingo de los Tsáchilas.

**Region**	**Province**	**Species**	**Ref.**
Andes	Pichincha	*interrogans*	[1,2]
	Cotopaxi	*interrogans*	[3]
Coast	Manabí	*borgpetersenii, interrogans, kirschneri, noguchii, santarosai, wolffii*	[4–10]
	Guayas	*interrogans*	[11–14]
	Santa Elena	*borgpetersenii, interrogans, kirschneri*	[15]
Amazon	Napo	*biflexa, meyeri, santarosai*	[16]
	Zamora Chinchipe	*borgpetersenii, interrogans*	[17]
Multiple*		*borgpetersenii, inadai, interrogans, kirschneri, santarosai, wolffii*	[18–21]

Table S3. Serovars of *Leptospira* identified in Ecuadorian provinces. Studies included those that specifically refer to Serovars. A total of 29 serovars have been identified in this review. *Multiple include Guayas, Los Ríos, Pichincha, Esmeraldas, and Santo Domingo de los Tsáchilas.

Region	Province	Serovars	Ref.
Andes	Pichincha	Bataviae, Bratislava, Canicola, Grippotyphosa, Hardjo, Icterohaemorrhagiae, Pomona, Sejroe	[1,22,23]
	Cotopaxi	Canicola, Icterohaemorrhagiae, Pomona, Sejroe, Tarassovi	[3]
	Loja	Autumnalis, Canicola, Hebdomadis, Patoc, Pomona	[24]
Coast	Manabí	Australis, Bataviae, Bratislava, Canicola, Copenageni, Copenhageni, Grippotyphosa, Hardjo, Icterohaemorrhagiae, Pomona, Sejroe, Tarassovi, Wolffi	[5,8–10]
	Guayas	Australis, Autumnalis, Ballum, Bataviae, Canicola, Celledoni, Copenhageni, Cynopteri, Djasiman, Grippotyphosa, Hardjo, Icterohaemorrhagiae, Javanica, Panama, Patoc, Pomona, Saxkoebing, Sejroe, Shermani, Tarassovi, Wolffi	[11–14,25,26]
	Santa Elena	Bataviae, Bratislava, Canicola, Grippotyphosa, Sejroe, Tarassovi	[15]
Amazon	Zamora Chinchipe	Australis, Bataviae, Canicola, Sejroe	[17]
Multiple*		Australis, Autumnalis, Babudieri, Bataviae, Borincana, Bratislava, Canicola, Castellonis, Celledoni, Copenhageni, Cynopteri, Djasiman, Grippotyphosa, Hardjo, Hebdomadis, Hyos, Icterohaemorrhagiae, Javanica, Panama, Patoc, Pomona, Pyrogenes, Saxkoebing, Sejroe, Shermani, Tarassovi, Wolffi	[18,21,27]

References supplementary material

1. Bravo M, León P. Primeras investigaciones sobre leptospirosis bovina en el Ecuador. Rev Ecuat Hig Med Trop. 1962;1: 23-44.
2. Gutierrez Ermel, Trueba Gabriel. Aislamiento de *Leptospira interrogans* de la orina de hembras bovinas en la provincia de Pichincha. Thesis. Universidad Central del Ecuador. 1986.
3. Lascano P, Arcos C, Lopez G, Mendez M, Soria M, Vallecillo M. Incidencia de leptospirosis en perros que habitan en zonas cercanas a la industria animal en Ecuador. Rev Ecuat Cienc Anim. 2018;1: 1-6.
4. Sosa A. Estudio piloto detección de *Leptospira* en el cantón Portoviejo (Manabí). Thesis. Universidad San Francisco de Quito. 2015. Available at: http://repositorio.usfq.edu.ec/handle/23000/4887.
5. Salinas A, Jorge C, Trueba G. Leptospirosis in febrile patients from neighboring parishes of Portoviejo (Ecuador). Quito, Ecuador. Universidad San Francisco de Quito. 2016. Available at: https://www.researchgate.net/publication/298788092_Leptospirosis_in_febrile_patients_from_neighboring_parishes_of_Portoviejo_Ecuador.
6. Barragan V, Chiriboga J, Miller E, Olivas S, Birdsell D, Hepp C. et. al. High *Leptospira* diversity in animals and humans complicates the search for common reservoirs of human disease in rural Ecuador. PLoS Negl Trop Dis. 2016a;13: e0004990.
7. Barragan V, Sahl JW, Wiggins K, Chiriboga J, Salinas A, Cantos NE, et al. 2016b. Draft genome sequence of the first pathogenic *Leptospira* isolates from Ecuador. Genome Announc. 2016b;4: e00271-16.
8. Burgos D, Perez M, Bulnes C, et al. Nivel de conocimiento de la leptospirosis bovina en la provincia Manabí, Ecuador. Rev Salud Anim. 2019;41. Available at: http://revistas.censa.edu.cu/index.php/RSA/article/view/1024.
9. Zambrano-Gavilanes MP, Pérez L, Guerrero Santana MV, et al. Seroprevalencia de anticuerpos contra *Leptospira* spp. en cerdos criados en Portoviejo, Ecuador. Rev Cubana Med Trop. 2020;72: e540.
10. Ruano MP, Burgos-Macıas DI, Bulnes CA, Zambrano MD, Sandoval HP, Falconí MA, et al. Seroprevalence and risk factors of bovine leptospirosis in the province of Manabí, Ecuador. Comp Immunol Microbiol Infect Dis. 2020;72: 101527.
11. Barrera-Sosa O. Un caso de leptospirosis ictero-hemorrágica. Rev Ecuat Hig Med Trop. 1970;27: 255-265.
12. Chedraui P, Gonzalez D, Casanova GSM, Gomnez A. Reporte de un caso de leptospirosis y embarazo. Rev Med. 2001;7: 75-78.
13. Campos J, Orlando A, Parraga K, Jurado E, Sans N, Yamchaliquin D. Circulación de leptospiras patogénicas en ratas noruegas (*Rattus norvergicus*) en la ciudad de Guayaquil. 2018. Available at: https://www.researchgate.net/publication/326066164.
14. Orlando SA, Perez A, Sanchez E, de la Cruz C, Rugel O. High seroprevalence of anti-*Leptospira* spp. antibodies in domestic and wild mammals from a mixed use rescue center in Ecuador: lessons for “One Health” based conservation strategies. One Health. 2020;10: 100140.

15. Orlando A, Paez-Martínez K, Sanchez E, de la Cruz C, Arcos F, Torres P, et al. Ultra high seroprevalence of anti-*Leptospira* spp. antibodies in racing horses from a breeding farm in Ecuador: need for One Health-based management. 2020.
16. Barragan VA, Mejia ME, Trávez A, Zapata S, Hartskeerl RA, Haake DA, et al. Interactions of *Leptospira* with environmental bacteria from surface water. Curr. Microbiol. 2011;62: 1802-1806.
17. Muyulema E. Estudio clínico epidemiológico de leptospirosis en hembras bovinas en edad reproductiva en el cantón El Pangui. Thesis. Escuela Superior Politécnica de Chimborazo. 2020. Available at: http://dspace.espoch.edu.ec/handle/123456789/14506.
18. Yépez W, Dávila A, Kinebuchi H. Actualización de leptospirosis en el Ecuador. Rev Ecuat Hig Med Trop 1979;32: 1-6.
19. Baquero MI, López N, Mejía ME, Trueba G. Evaluation of a polymerase chain reaction for the diagnosis of leptospirosis in cattle. Open Vet Sci J. 2010;4: 31-35.
20. Chiriboga J, Barragán V, Arroyo G, Sosa A, Birdsell DN, España K, et al. High prevalence of intermediate *Leptospira* spp. DNA in febrile humans from urban and rural Ecuador. Emerg Infect Dis. 2015;21: 2141-2147.
21. Mendoza RJ. Situación epidemiológica de la leptospirosis humana en la región Costa del Ecuador, 2005-2012. Thesis. Univerisidad de Guayaquil. 2015. Available at: http://repositorio.ug.edu.ec/handle/redug/9838.
22. Maldonado A. Estimación de la población de caninos en mercados del centro de Quito mediante un estudio demográfico y determinación del estatus zoosanitario en la relación a *Leptospira*, *Dipylidum caninum* y *Toxocara canis*. Thesis. Universidad de las Américas. 2018. Available at: http://dspace.udla.edu.ec/handle/33000/8856.
23. Chiriboga C. Evaluación del estatus sanitario de bovinos, ovinos, caninos y porcinos respecto a leptospirosis mediante la prueba MAT en la granaja experimental UDLA Nono, Quito. Thesis. Universidad de las Américas. 2019. Available at: https://dspace.udla.edu.ec/bitstream/33000/11827/1/UDLA-EC-TMVZ-2019-40.pdf.
24. Pinta D, Perez G. Determinación de la prevalencia de leptospirosis en pacientes caninos atendidos en el hospital docente veterinario "César Augusto Guerrero". Thesis. Universidad Nacional de Loja. 2021. Available at: https://dspace.unl.edu.ec//handle/123456789/23821.
25. Gamboa AA, Vasco L, Espinel M, et al. Difficulties in the differential diagnosis of dengue and leptospirosis in Guayaquil. Av En Cienc E Ing. 2013;5: B10-B13.
26. Torres P, Miño G. Leptospirosis severa en pediatría. Informe de un caso. 2019. [Internet]. Available at: https://www.medicosecuador.com/espanol/articulos_medicos/leptospirosis-severa-pediatria.htm.
27. Chávez-Vásquez J. Primeros estudios epizootiológicos de leptospirosis porcina en el Ecuador. Rev Ecuat Hig Med Trop. 1985;35: 49-54.
